# Is there a difference in pelvic and femoral morphology in early periprosthetic femoral fracture in cementless short stem total hip arthroplasty via an anterolateral approach?

**DOI:** 10.1186/s10195-024-00795-x

**Published:** 2024-11-04

**Authors:** Matthias Luger, Sandra Feldler, Clemens Schopper, Tobias Gotterbarm, Christian Stadler

**Affiliations:** 1 Department for Orthopedics and Traumatology, Kepler University Hospital GmbH, Krankenhausstrasse 9, 4020 Linz, Austria; 2 Johannes Kepler University Linz, Altenberger Strasse 69, 4040 Linz, Austria

**Keywords:** Short stem, THA, Total hip arthroplasty, Periprosthetic fracture, PFF, Femoral canal

## Abstract

**Background:**

The pelvic and femoral morphology are associated with the occurrence of early periprosthetic femoral fractures (PFFs) in cementless total hip arthroplasty (THA). Differences exist depending on the performed approach and implanted stem design. Therefore, this study was conducted to analyze the pelvic and femoral morphology in cementless short stem THA via a minimally-invasive (MIS) anterolateral approach.

**Methods:**

A retrospective, single-center, multi-surgeon, comparative propensity-score matched study of a cohort of 1826 short stem THAs was conducted. A total of 39 PFFs within the first 90 days after surgery was matched on a 2:1 ratio to non-fracture patients. The morphology of the proximal femur was analyzed with canal flare index (CFI), canal-calcar ratio (CCR), canal-bone ratio (CBR), morphological cortical index (MCI), and femoral cortical index (CI). The pelvic morphology was analyzed with ilium–ischial ratio (IR), distance anterior superior iliac spine to the tip of the greater trochanter (AGT). Both groups were analyzed regarding several parameters for femoral and pelvic morphology in non-parametric testing and univariate regression analysis.

**Results:**

A significantly higher AGT was detected in the fracture group (104.5 mm ± 18 versus 97.4 mm ± 9.8; *p* = 0.016). All other femoral and pelvic parameters did not differ between both groups, also when compared depending on the Vancouver type of the PFF.

**Conclusions:**

The morphology of the proximal femur and the pelvis do not differ in several radiological parameters in patients sustaining a PFF in cementless short stem THA via an anterolateral approach compared with matched non-fracture group. The findings are controversial to other studies with different stem types and approaches. Future studies should focus on analyzing the influence of the pelvic geometry and the shape of the proximal femur in the occurrence of PFFs in different approaches with the same stem type and vice versa.

*Level of Evidence* Level III case-controlled study

## Introduction

Cementless fixation in total hip arthroplasty (THA) is associated with an increased risk for periprosthetic femoral fractures (PFFs) [1]. Especially single-wedge and double-wedge (fit-and-fill) femoral implants show an up to threefold increase in PFF rates compared with anatomical, fully coated, and tapered/rounded stems [1]. The occurrence of a PFF is multifactorial with several known risk factors, such as implant design [1–3], surgical approach [1–3], bone quality or the presence of osteoporosis [4, 5], implant position [4, 5], increasing age [4, 5], sex [4–6], and secondary OA owing to rheumatoid arthritis [1, 5].

Minimally invasive (MIS) approaches to the hip have been increasingly performed in recent years as they allow a faster recovery with decreased pain levels owing to reduced damage of soft tissue [7–9]. However, MIS approaches in combination with standard length straight stems are associated with a more difficult and challenging femoral exposure and broaching of the femoral canal, which may increase the risk of malpositioning and nonoptimal sizing of the femoral component leading to an increased risk for PFFs [10, 11]. With increasing popularity of MIS approaches, the use of shorter cementless femoral stems has been increasingly adopted [2, 12]. The shorter geometry of these types of stems may allow an easier introduction of the broach into the femoral canal hence facilitating an easier femoral preparation [2, 12]. Cementless short stems area associated with a reduced rate of PFFs in MIS THA [2, 3]. Dietrich et al. [2] report a reduced rate of PFFs of 1.6% in direct anterior approach (DAA) in using cementless short stems compared with 6.8% for cementless straight stems in a 2-year study period (*p* = 0.027). Luger et al. [3] report a reduced rate of PFFs with 1.7% in short stem THA compared with 3.2% in straight stem THA within the 1st year after index surgery (*p* = 0.015). The reduced rates of PFFs might be associated with an easier insertion of short stems while broaching in MIS THA [2, 3].

The influence of the pelvic and femoral morphology on the occurrence of PFFs is not fully understood [11]. McGoldrick et al.[11] analyzed differences in the pelvic and femoral morphology in MIS THA with a cementless short stem implanted via a DAA in a propensity score matched analysis. They reported a fracture risk 29 times higher in patients with a canal flare index (CFI) > 3.17 and an ilium-ischial ratio > 3. Bigart et al.[13] examined the influence of the femoral morphology on the occurrence of PFFs in cementless straight stem THA in a propensity score matched analysis. Patients sustaining an acute early PFF showed thinner distal cortices and a decreased meta-diaphyseal taper [13].

The morphology of the proximal femur and the pelvis can play a significant role in the occurrence of PFFs in cementless short stem [11] and straight stem [13, 14] THA. The current data allows to conclude several pelvic and femoral morphological differences only for certain stem types and approaches. Therefore, this propensity score matched study was conducted to perform an analysis on the pelvic and femoral morphological differences in MIS THA using a MIS anterolateral approach in supine positioning with a cementless curved short stem in patients sustaining an acute PFF within the first 90 days compared with a matched non-fracture group.

## Material and methods

### Study design

A retrospective, single-center, multi-surgeon, comparative propensity-score matched study of a cohort of 1826 short stem THAs at a single large academic center between 2011 and 2021 was conducted.

### Cohort

The institutional electronic database was used to obtain information on patients who underwent primary THA with a cementless short stem (Fitmore® hip stem; ZimmerBiomet, Warsaw, IN, USA) implanted via a minimally-invasive anterolateral approach in supine positioning [15] between 1 January 2011 and 31 December 2021. Diagnoses for inclusion were defined as primary osteoarthritis of the hip, osteonecrosis or mild hip dysplasia (Crowe 1). All cases of secondary forms of osteoarthritis and all cases with previous surgeries were excluded. In total, 1826 THAs met the inclusion criteria for screening for a PFF between the first 90 days of index surgery and for the propensity score matching.

The study was approved by the institutional review board (no.: 1232/2022). Because of the retrospective anonymized evaluation of preexisting medical records, an informed consent was not required. All procedures performed in studies involving human participants were in accordance with the ethical standards of the institutional and/or national research committee and with the 1964 Helsinki declaration and its later amendments or comparable ethical standards.

### Propensity score matching

In total, 1826 THAs cases were screened for the occurrence of PFFs. In the study period 39 PFFs (2.1%) within the first 90 days after index surgery occurred. The type of fracture was recorded by localization of the fracture and by classification according to the Vancouver classification for intra- and postoperative PFFs [16–18]. These cases were matched on a two-to-one ratio with 78 nonfracture cases as a control group. The propensity score matching was performed including several variables as risk factors for PFFs such as age, sex, Dorr classification, body mass index (BMI), side, femoral component offset, surgeon’s experience, American Society of Anesthesiologists (ASA) Score, and indication for surgery. Owing to the retrospective cohort these well-known parameters and risk factors were included in the matching process to reduce bias by matching based on patient-specific, implant-specific and surgical factors. A 2:1 propensity score matching using the caliper technique with a caliper set at 0.2 was performed to create a 2:1 distribution of non-fracture and fracture patients. The patient demographics of the fracture group and the control group are given in Table 1. A distribution of comorbidities and drugs potentially influencing the fracture risk is given in Appendix 1 for both matched groups.

**Table 1:** Patient demographics for fracture and non-fracture group

Patient Demographics	Fracture (*n* = 39)	Non-fracture (*n* = 78)	*p* Value
Sex			
Female	29 (74.4%)	48 (61.5%)	0.216
Male	10 (25.6%)	30 (38.5%)	
Age at operation (in years)	74.4 ± 9.3	74.4 ± 11	0.768
Diagnosis			
Primary OA	30 (76.9%)	66 (84.6%)	0.185
ON	8 (20.5%)	7 (9.0%)	
Hip dysplasia	1 (2.6%)	5 (6.4%)	
Side			
Left	15 (38.5%)	32 (41%)	0.843
Right	24 (61.5%)	46 (59%)	
ASA score			
1	3 (7.7%)	4 (5.1%)	0.745
2	16 (41%)	36 (46.2%)	
3	20 (51.3%)	36 (46.2%)	
4	0 (0.0%)	2 (2.6%)	
Surgical experience			
Consultant	33 (84.6%)	60 (76.9%)	0.467
Resident	6 (15.4%)	18 (23.1%)	
BMI (kg/m^2^)	28.1 ± 5.1	27.6 ± 5.9	0.379
Height (cm)	165.9 ± 8.8	164.3 ± 9.5	0.322
Weight (kg)	77.5 ± 16.7	75.1 ± 19.5	0.338
Dorr classification			
A	4 (10.3%)	8 (10.3%)	1.000
B	28 (71.8%)	56 (71.8%)	
C	7 (17.9%)	14 (17.9%)	
Offset option			
A 140°	5 (12.8%)	14 (17.9%)	0.581
B 137°	27 (69.2%)	44 (56.4%)	
B extended 129°	7 (17.9%)	18 (23.1%)	
C 127°	0 (0.0%)	2 (2.6%)	

### Surgical procedure and implants

In total, 34 surgeons performed the surgeries. All cases by consultants and resident were included. All consultants performed at least 50 arthroplasty surgeries per year. Residents performed the surgeries with the presence of a consultant. Full weight-bearing was allowed immediately on the day of surgery. Minimally invasive anterolateral approach was performed in supine positioning [15], by bluntly dissecting the Watson-Jones interval between tensor fasciae latae and gluteus medius. A skin incision was centered over the greater trochanter. After subcutaneous dissection an incision at the border between the tensor fasciae latae and the tractus iliotibilias was performed. Then, the Watson-Jones interval between tensor fasciae latae and gluteus medius was bluntly dissected. A capsulectomy was performed in each case. Exposure of the femur was conducted with diathermia. The adjacent superior capsule in the piriformis’ fossa and the inferior hip capsule close to the lesser trochanter are released in order to mobilize the femur sufficiently to insert a specifically design MIS-retractor posterolateral to spare the gluteus medius. The knee is flexed at 90° and crosses the other leg underneath.

A cementless short stem (Fitmore^®^ hip stem, ZimmerBiomet, Warsaw, IN, USA) was used in every case. Fitmore^®^ hip stem is a titanium alloy stem (Ti Al6V4) that has a porolock Ti-VPS coating in the proximal part to enhance bone ingrowth and is available in four different neck angle options (127°, 129°, 137°, and 140°) The stem has a triple-tapered design to achieve press-fit fixation at the metaphyseal/diaphyseal level [19]. A cementless titanium press-fit cup with or without screws (Allofit^®^/-S, ZimmerBiomet, Warsaw, IN, USA) or two types of cementless threaded cups (Alloclassic CSF^®^/Alloclassic Variall^®^, both ZimmerBiomet, Warsaw, IN, USA) were used. In all cases a digital preoperative template of the acetabular and femoral component was conducted by the surgeon using mediCAD® version 5.1 (Hectec GmbH, Altdorf, Germany) on digital low-centered anteroposterior (AP) radiographs of the pelvis.

### Radiographic measurements

**Fig. 1:**
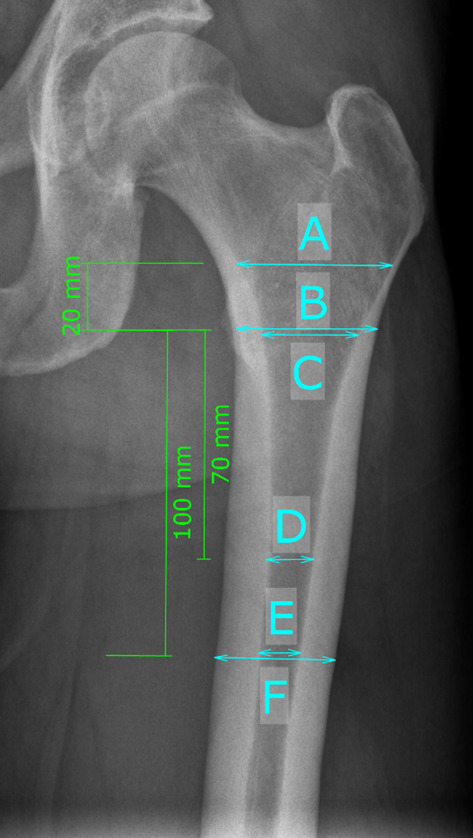
Schematic measurements of the parameters of the proximal femur: canal flare index (CFI) = A/E; canal-calcar ratio (CCR) = E/C; canal-bone ratio (CBR) = E/F; morphological cortical index (MCI) = B/D; femoral cortical index (CI) = (F-E)/F

**Fig. 2:**
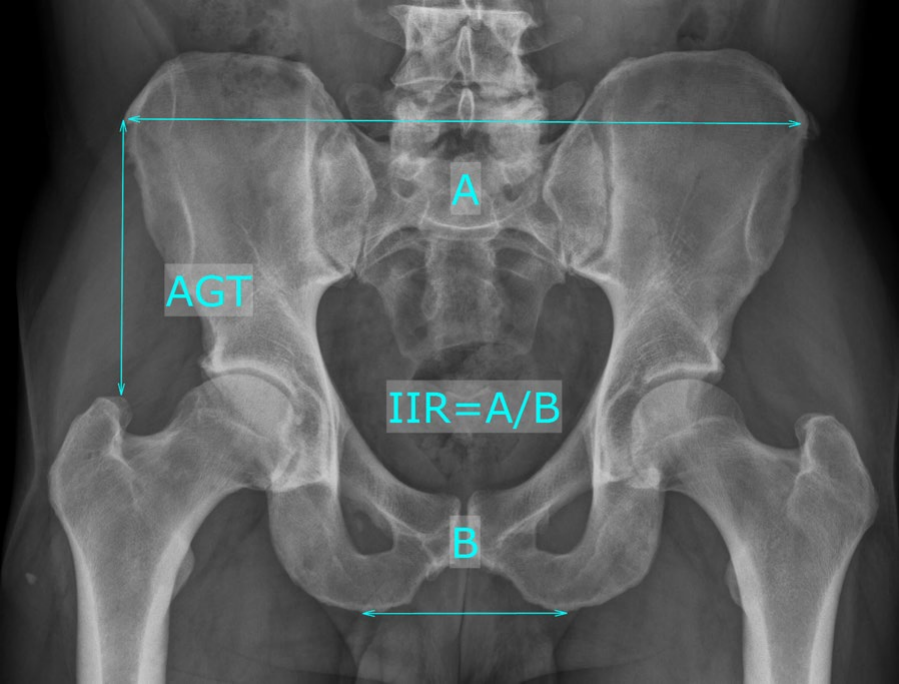
Pelvic morphological parameters on an anteroposterior pelvic radiograph: ilium–ischial ratio (IIR) = A/B; distance anterior superior iliac spine to the tip of the greater trochanter (AGT)

The measurements of the morphological parameters of the proximal femur and pelvis were measured on digital low-centered AP radiographs of the pelvis using mediCAD® version 5.1 (Hectec GmbH, Altdorf, Germany). Radiographs were taken with the patient in standing position and with both legs in 15° internal rotation and the central beam was directed on the symphysis pubis with a film focus distance of 1.15 m. The magnification error was corrected with a 25 mm radiopaque ball as the templating reference at the level of the hip between the legs or within the field of view. Proximal femoral geometry was analyzed using previously described radiographic parameters [11, 13]. The parameters for analyzing the morphology of the proximal femur include the canal-flare index (CFI) [20], the canal-calcar ratio (CCR) [21], canal-bone ratio (CBR) [22], the morphological cortical index (MCI) [23], and the femoral cortical index (CI) [24], see Fig. 1. For analysis of the pelvis the ilium–ischial ratio (IIR) [11] and the distance from the anterior superior iliac spine (ASIS) of the tip of the greater trochanter (AGT) [11] were measured, see Fig. 2. The measurements of all parameters are described in detail in Table 2 [13, 25]. All measurements were conducted by two observers (S.F., C.S.). Intra- and interobserver correlation coefficients (ICC) were obtained with excellent agreements (ICC 0.758–0.974).

**Table 2:** Radiographic measurements

Radiographic measurement	Acronym	Definition
Canal flare index	CFI	Ratio between metaphyseal measured 2 cm proximal to the lesser trochanter divided by the with of the endosteal canal at the level of the lesser trochanter
Canal-calcar ratio	CCR	Ratio of the endosteal diameter 10 cm below the lesser trochanter to the endosteal diameter at the level of the lesser trochanter
Canal-bone ratio	CBR	Width of the endosteal canal divided by the width of the external
Morphological cortical index	MCI	Ratio of the external diameter at the level of the lesser trochanter to the endosteal diameter at a point 7 cm below the lesser trochanter
Femoral cortical index	CI	Ratio of cortical width minus endosteal width to cortical width at a level of 100 mm below the tip of the lesser trochanter
Ilium–ischial ratio	IIR	Ratio of the width between both anterior superior iliac spines and the width of both ischial tuberosities
Distance anterior superior iliac spine to the tip of the greater trochanter	AGT	Distance from the anterior superior iliac spine to the tip of the greater trochanter

**Fig. 3:**
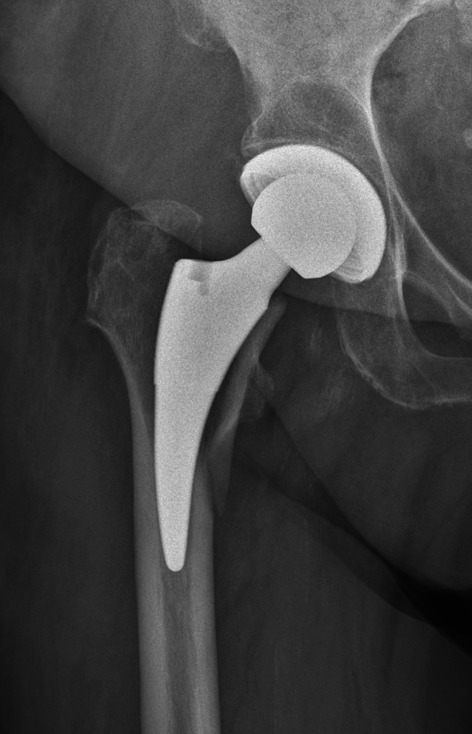
Illustrative case of a postoperatively detected periprosthetic femoral fracture type Vancouver B2

### Data analyses

Descriptive analyses were performed for patient demographics. Shapiro–Wilk tests for normality were performed to determine whether continuous data were normally distributed. As the variables were not normally distributed, a Fisher’s Exact test was used for categorical variables and a Mann–Whitney *U*-test was performed for comparison of continuous data. A univariate regression analysis was performed for analyzing the different femoral and pelvic parameters as a risk factor for the occurrence of a PFF. All statistical analyses were performed using SPSS statistical software (version 29.0, SPSS Inc, Armonk, NY). Statistical significance was set at *p* < 0.05.

## Results

In total, 39 PFFs were detected in the study period, see Table 3. Of these, 11 PFFs (28.2%) were detected intraoperatively and 28 PFFs (71.8%) were detected postoperatively, see Table 3. Vancouver B PFFs were the most common PFFs in the study group with 66.6%. Of these PFFs, the most common type was a postoperatively detected Vancouver B fracture (53.8%). Table 3 shows the distribution of all PFFs according to the Vancouver classification. Appendix 2 shows the fracture type, Vancouver classification, cause of fracture, revision, and treatment of PFFs in detail. Figure 3 illustrates a typical Vancouver B2 fracture of the Fitmore® hip stem.

**Table 3:** Type of fractures, intra- or postoperative occurrence and distribution according to the Vancouver classification

Intraoperative			Postoperative		
Total	11	28.2%	Total	28	71.8%
Vancouver classification			Vancouver classification		
*Vancouver A*	6	15.4%	*Vancouver A*	7	17.9%
A1	0	0.0%	AG	7	17.9%
A2 LT	4	10.3%			
A2 GT	2	5.1%			
A3 LT	0	0.0%			
A3 GT	0	0.0%			
*Vancouver B*	5	12.8%	*Vancouver B*	21	53.8%
B1	0	0.0%	B1	1	2.6%
B2	5	12.8%	B2	20	51.2%
B3	0	0.0%	B3	0	0.0%
*Vancouver C*	0	0.0%	*Vancouver C*	0	0.0%
C1	0	0.0%			
C2	0	0.0%			
C3	0	0.0%			

All femoral parameters did not show any statistical significance between both groups, see. Table 4. The pelvic geometry did only differ significantly in the AGT between both groups (*p* = 0.024), see Table 4. The separate analysis of both stem groups depending on the Vancouver classification did not show any differences for Vancouver A and Vancouver B PFFs for both stem types, see Table 5.

**Table 4:** Pelvic and femoral morphological parameters between fracture and non-fracture patients

	Fracture (*n* = 39)	Non-fracture (*n* = 78)	*p* Value
AGT	104.5 ± 18	97.4 ± 9.8	**0.016**
Ilium–ischial ratio	2.95 ± 0.58	2.93 ± 0.47	0.613
Canal flare index	3.34 ± 0.55	3.37 ± 0.49	0.788
Canal–calcar ratio	0.49 ± 0.07	0.49 ± 0.06	0.892
Canal–bone ratio	0.43 ± 0.05	0.44 ± 0.05	0.851
Morphological cortical index	2.69 ± 0.39	2.63 ± 0.37	0.184
Cortical index	0.57 ± 0.05	0.56 ± 0.05	0.851

Significant *p*-values are written in bold.

**Table 5:** Pelvic and femoral morphological parameters between Vancouver A and B fractures

	Fracture (*n* = 39)	Vancouver A (*n* = 13)	Vancouver B (*n* = 26)	*p* Value
AGT	104.5 ± 18	106.7 ± 27.3	103.5 ± 11.5	0.502
Ilium–ischial ratio	2.95 ± 0.58	2.68 ± 0.39	3.08 ± 0.61	0.051
Canal flare index	3.34 ± 0.55	3.28 ± 0.58	3.37 ± 0.54	0.512
Canal–calcar ratio	0.49 ± 0.07	0.52 ± 0.1	0.49 ± 0.07	0.363
Canal–bone ratio	0.43 ± 0.05	0.43 ± 0.53	0.43 ± 0.05	0.835
Morphological cortical index	2.69 ± 0.39	2.72 ± 0.46	2.68 ± 0.37	0.687
Cortical index	0.57 ± 0.05	0.57 ± 0.05	0.57 ± 0.05	0.848

The univariate analysis showed a significant increased odds ratio (OR) for the occurrence of a PFF with an increased AGT [OR 1.050; 95% confidence interval (CI) 1.010–1.092; *p* = 0.015]. All other parameters did not show any significantly increased OR for the occurrence of a PFF. Table 6 shows the detailed results for the univariate regression analysis.

**Table 6:** Univariate analysis for pelvic and femoral morphological parameters for the risk of periprosthetic femoral fracture

	Odds ratio	95% confidence interval	*p* Value
AGT	1.050	1.010–1.092	**0.015**
Ilium–ischial ratio	1.088	0.507–2.334	0.828
Canal flare index	0.903	0.424–1.926	0.792
Canal–calcar ratio	12.333	0.036–4172.268	0.398
Canal–bone ratio	0.183	0.000–308.860	0.654
Morphological cortical index	1.608	0.569–4.543	0.370
Cortical index	5.460	0.003–9209.163	0.654

Significant *p*-values are written in bold.

## Discussion

In the current study, differences in the geometry of the proximal femur and the pelvis were compared in patients sustaining an early PFF to non-fracture patients in cementless short stem THA implanted via a MIS anterolateral approach. The results do not show significant differences in the morphology of the proximal femur and the pelvis also analyzed in subgroups for Vancouver A and B PFFs as well as in the univariate regression analysis.

Studies investigating the differences of the proximal femur and the pelvic geometry have been recently published for cementless short and straight stem THA [11, 13, 14]. McGoldrick et al. [11] published data on THA with a type 4 short stem according to Khanuja et al. [26] implanted via a DAA. In the fracture group a significantly higher CFI was found, with 3.69 compared with 2.93 in the non-fracture group (*p* < 0.001) [11]. The risk of a PFF was 29 times higher in patients with a CFI > 3.17 [11]. The Fitmore® hip stem investigated in the presented study is also classified as a type 4 short stem according to Khanuja et al. [26]. The femoral parameters in the fracture group for the Fitmore® stem implanted through a MIS anterolateral approach are comparable to the results reported by McGoldrick et al. [11]. However, unlike the non-fracture group in the study by McGoldrick et al., the non-fracture group did not show any significant differences [11]. This might indicate that different stems and approaches might be associated with differences in the femoral morphology in cases of early PFFs. Although both stems are classified in the same category [26], they do not result in the same values in fracture and non-fracture patients regarding the proximal femur. The difference between both studies might be more associated with the approach rather than the stem design. The different necessity for femoral release and also the different insertion of the broach might be factor for these varying results. Although the DAA and the MIS anterolateral approach are both anterior-based approaches in supine positioning, there might be possibility of different risk for PFFs regarding the anatomy of the proximal femur, that results from the slight differences in performing these approaches.

Bigart et al.[13] report a significantly lower CFI of 3.05 in the fracture group compared with 3.28 in a matched control group in cementless straight stem THA. Griffiths et al. [14] report opposing data in for cementless THA via a DAA compared with McGoldrick et al. [11]. A lower CFI was predictive for an early PFF with a mean CFI of 2.75 in the fracture group compared with 3.2 in the control group [14]. The results by Bigart et al. [13] and Griffiths et al. [14] show an increased risk of PFFs in cementless straight stem THA in patients with thinner distal cortices and a decreased meta-diaphyseal taper. However, both studies [11, 14] mainly include Vancouver B or Vancouver B2 PFFs, while McGoldrick et al. [11] included all fracture types according to the Vancouver classification. Our study also cannot reproduce the findings as we analyzed Vancouver A and B fractures separately, as in comparable studies [13, 14]. Bigart et al. [13] reported a matched cohort in a comparable 2:1 propensity score matching; however, they showed significant differences in the included approaches and stem types between the fracture and non-fracture groups. Griffiths et al. [14] reported only on PFFs in Vancouver B fractures in THA with a DAA with a single stem type. Therefore, comparisons between different stem types and approaches might not be possible owing to different characteristics depending on the stem geometry and the technical aspects of the different approaches. Additionally, the different patient demographics, implants, and approaches in the propensity score matching might be a reason for the different results.

Additionally, the geometry of the pelvis might contribute to an increased risk of PFFs [11]. McGoldrick et al. [11] report a higher IIR as well as a lower AGT in patients sustaining a PFF in short stem THA. In the presented study the AGT was significantly higher in the fracture group, while the IIR show no significant difference between both stem types. McGoldrick et al. [11] reported a significantly increased risk with an IIR > 3. In the presented study the IIR was reported with values < 3 for fracture and non-fracture patients. However, the analysis depending on the Vancouver type of the PFF showed an IIR of 3.08 in Vancouver B fractures with almost significant difference compared with Vancouver A PFFs (*p* = 0.051). This might indicate an influence of the IIR on the occurrence of PFFs in short stem THA via a MIS anterolateral approach. However, the presented data does not reflect a definitive significant influence compared with short stem THA using the DAA [11]. Interestingly, the only significant parameter found in the presented study was the significantly higher AGT in the fracture group, which is controversial to the findings for short stem THA via the DAA, which shows a significantly lower AGT in patients with fractures [11]. The study groups by McGoldrick et al. [11] and the present study have also been matched on the offset option of the used stem. Similarly, Sun et al. [27] also found a lower AGT to be a risk factor for the occurrence of PFFs in DAA in lateral decubitus position using a conventional femoral stem. However, a similar stem length might not pose the same risk regarding the pelvic geometry and especially the distance between the anterior superior iliac spine (ASIS) and the greater trochanter. Therefore, the slightly different insertion of the broach in the different approaches might be the possible factor for the controversial results for the differences in the AGT between two similar stem types with similar stem length. A lower AGT might be more relevant in DAA as it could impair inserting the broach entering the femoral canal for preparation in a straight line, while a higher AGT might impair broaching in anterolateral approach as broaching as entering the femoral approach is not as steep as in DAA.

Limitations of the study include the retrospective collective and study design. However, a possible selection bias was addressed with a propensity score matching, that included a high number of different variables. Therefore, a possible selection bias was addressed with a thorough propensity score matching process. Additionally, a possible selection bias might be present, that patients with a high risk were not elected for short stem THA in the first place. However, at the authors’ institution, cemented THA was rarely performed for a primary THA. Additionally, after the learning curve, the short stem has been used irrespective of patients age, sex, or indication and has become the standard implant for primary THA at the authors’ institution. Therefore, we report a case series with a high number of THAs with a broad range of patients. A further limitation is the performed measurement on anterior–posterior radiographs. Computed tomography (CT) might provide a higher quality in imaging and would also report a higher number of occult early PFFs that might be undetected on standard x-rays. However, CT imaging would result in a significantly increased exposure to radiation for the patient. Additionally, the study methods are well described in literature and were used in comparable studies, giving it sufficient comparability to other studies. The measurements on plain radiographs are also easy to perform and pose therefore it is possible to repeat the study design for other stem types or approaches.

## Conclusions

The morphology of the proximal femur and the pelvis do not differ in several radiological parameters in patients sustaining a PFF in cementless short stem THA via an anterolateral approach compared with matched non-fracture group. The findings are controversial to other studies with different stem types and approaches. Future studies should focus on analyzing the influence of the pelvic geometry and the shape of the proximal femur in the occurrence of PFFs in different approaches with the same stem type and vice versa.

## Data Availability

Data and materials are available on request.

## Appendix 1

See Table 7

**Table 7:** Distribution of different comorbidities and medication of potentially influencing fracture risk

	Fracture (*n* = 39)	Non-fracture (*n* = 78)	*p* value
Rheumatoid arthritis	3 (7.7%)	5 (6.4%)	0.796
Osteoporosis	3 (7.7%)	9 (11.5%)	0.518
COPD/Asthma bronchiale	8 (20.5%)	10 (12.8%)	0.277
Chronic kidney disease	3 (7.7%)	14 (17.9%)	0.138
Calcium substitution	3 (7.7%)	16 (20.5%)	0.076
Vitamin D substitution	4 (10.3%)	14 (17.9%)	0.277
Bisphosphonate	0 (0.0%)	5 (6.4%)	0.106
Teriparatid	2 (2.6%)	1 (1.3%)	0.614
Denosumab	0 (0.0%)	1 (1.3%)	0.478
Corticosteroids	3 (7.7%)	3 (3.8%)	0.374

## Appendix 2

See Table 8

**Table 8:** Fracture type, Vancouver classification, cause of fracture, revision and treatment of periprosthetic femoral fractures (PFFs)

Short stem					
Fracture type	Vancouver classification	Cause of fracture	Time of fracture	Revision (yes/no)	Treatment
Intraoperatively (*n* = 11)					
Greater trochanter	GT A2	Retraction with Hohmann retractor		N	One cerclage cable
Greater trochanter	GT A2	Unknown		N	One cerclage cable
Medial cortex	LT A2	Stem insertion		N	One cerclage cable
Medial cortex	LT A2	Stem insertion		N	One cerclage cable
Medial cortex	LT A2	Stem insertion		N	One cerclage cable
Medial cortex	LT A2	Stem insertion		N	One cerclage cable
Medial cortex	B2	Stem insertion		N	Two cerclage cables, monoblock straight stem
Medial cortex	B2	Stem insertion		N	One cerclage cable
Lateral cortex	B2	Stem insertion		N	Two cerclage cables
Lateral cortex	B2	Stem insertion			Conservatively
Lateral cortex	B2	Stem insertion		Y	Intraoperatively treated with one cerclaged cable, failed in the follow-up leading to stem exchange to monoblock revision stem, three cerclage cables
Postoperatively (*n* = 28)					
Greater trochanter	AG	No history of fall	3	N	Conservatively
Greater trochanter	AG	No history of fall	4	N	Conservatively
Greater trochanter	AG	No history of fall	4	N	Conservatively
Greater trochanter	AG	No history of fall	4	N	Conservatively
Greater trochanter	AG	Avulsion fracture	4	N	Conservatively
Greater trochanter	AG	Avulsion fracture	6	N	Conservatively
Greater trochanter	AG	Avulsion fracture	10	N	Conservatively
Medial cortex	B1	No history of fall	70	N	Conservatively
Medial cortex	B2	No history of fall	3	J	Three cerclage cables, monoblock revision straight stem
Medial cortex	B2	No history of fall	5	J	Four cerclage cables, monoblock revision straight stem
Medial cortex	B2	No history of fall	5	J	Three cerclage cables, monoblock straight stem
Medial cortex	B2	No history of fall	8	J	Two cerclage cables, cemented monoblock straight stem
Medial cortex	B2	No history of fall	9	J	Three cerclage cables, monoblock revision straight stem
Medial cortex	B2	No history of fall	10	J	Two cerclage cables, monoblock straight stem
Medial cortex	B2	No history of fall	16	J	Three cerclage cables, monoblock straight stem
Medial cortex	B2	No history of fall	17	J	Three cerclage cables, monoblock straight stem
Medial cortex	B2	Fell while walking	20	J	One cerclage cable, monoblock straight stem
Medial cortex	B2	Fell while walking	25	J	Three cerclage cables, monoblock revision straight stem
Medial cortex	B2	Fell while walking	63	J	Three cerclage cables, modular revision straight stem
Lateral cortex	B2	No history of fall	3	J	Three cerclage cables, monoblock revision straight stem
Lateral cortex	B2	Fell while walking	5	J	Five cerclage cables, monoblock revision straight stem
Lateral cortex	B2	No history of fall	16	J	Three cerclage cables, monoblock straight stem
Lateral cortex	B2	No history of fall	17	J	Three cerclage cables, cemented monoblock revision straight stem
Lateral cortex	B2	Fell while walking	18	J	Three cerclage cables, monoblock straight stem
Lateral cortex	B2	Fell while walking	70	J	Three cerclage cables, monoblock straight stem
Lateral cortex	B2	Fell while walking	85	J	Four cerclage cables, monoblock revision straight stem
Lateral cortex	B2	Fell while walking	90	J	Three cerclage cables, monoblock revision straight stem
Spiral fracture	B2	Fell while walking	35	J	Four cerclage cables, monoblock revision straight stem
